# Confidence-Gated Triage: Coupling Drug–Target Affinity and ADME-T Predictions to Prioritise Compounds for Docking

**DOI:** 10.3390/ph19091445

**Published:** 2026-09-11

**Authors:** Gozde Yalcin Ozkat

**Affiliations:** Department of Bioengineering, Faculty of Engineering & Architecture, Recep Tayyip Erdogan University, Rize 53100, Turkey; gozde.yalcin@erdogan.edu.tr

**Keywords:** chemoinformatics, virtual screening, drug–target affinity, ADME-T prediction, machine learning

## Abstract

**Background/Objectives**: Molecular docking and molecular dynamics are accurate but computationally expensive, so the compounds entering them must be chosen well. The present study proposes CADT, a confidence-gated affinity–ADME-T docking-triage cascade that decides which compounds are worth docking. **Methods**: The gate combines an ensemble estimate of drug–target affinity with its epistemic uncertainty and an applicability-domain check. Predicted absorption, distribution, metabolism, excretion, and toxicity (ADME-T) developability is added as a soft flag. All components were trained on openly licensed Therapeutics Data Commons data. Ranking was assessed on the DAVIS and KIBA kinase panels and on BindingDB $K_{d}$, under three split protocols over five seeds. The routing decision was then examined against molecular docking, in which 407 compound–target pairs were docked into six withheld kinases. **Results**: A Morgan-fingerprint gradient-boosting model reached a concordance index of 0.866±0.006, with 0.813 for unseen targets and 0.720 for unseen drugs. Across eight ADME-T endpoints, the area under the ROC curve ranged from 0.65 to 0.91. On the cold-target split the cascade reduced the compounds sent to docking by 86% while retaining 61% of the true strong binders. Docking measured that reduction at 85%, and at an equal budget, the gate enriched true binders more than the docking score itself. **Conclusions**: A transparent pre-screen can prioritise compounds ahead of structure-based calculation at a fraction of its cost. However, the uncertainty and applicability-domain terms act as an abstention mechanism rather than an accuracy gain, and that abstention is not free.

## 1. Introduction

Machine learning (ML) is changing how drugs are discovered. Data-driven models now speed up target identification, molecular design, and property prediction across the pipeline [1,2,3,4]. Furthermore, they support artificial intelligence (AI)-driven pharmaceutical technology, design, and delivery [5,6,7,8,9]. The shift is not limited to pharmacology. Learned models match or beat classical methods in vibration-based anomaly detection for unmanned aerial vehicles [10,11]. Similarly, they match or beat them in laser-weld monitoring [12] and geotechnical assessment [13]. Recent reviews warn that many reported gains do not survive prospective testing, and that realistic evaluation and reproducibility, not headline accuracy, earn trust [14,15]. One practical answer treats cheap learned models not as replacements for physics-based simulation, but as a fast filter placed in front of it. Such a filter sorts large libraries before the costly stages of a structure-based campaign [16,17].

In early-stage drug discovery, structure-based methods stay central to prioritising and rationalising candidate ligands: molecular docking, molecular dynamics (MD) simulation, and end-point free-energy calculations such as MM/PB(GB)SA. Such workflows have been applied to GSK3β in Alzheimer’s disease [18] and to the BRPF1 bromodomain in leukaemia [19]. They have also addressed EGFR in head-and-neck cancer [20] and other neurodegeneration- and cancer-related proteins [21,22]. Moreover, they support therapeutic-target identification through data-driven genomic analysis [23]. They are physically grounded and interpretable, but expensive, and they scale poorly to the large libraries now screened. ML is therefore often paired with them. Examples include deep generative models coupled to docking and MD for SARS-CoV-2 inhibitor discovery [24]. Similarly, deep learning has been combined with structure-based virtual screening against novel oncology targets [25]. ML surrogates have also guided experimental optimisation [26].

Two prediction tasks matter most for a docking-driven workflow. The first is drug–target affinity (DTA) prediction, the ML analogue of docking. Given a drug (SMILES string) and a target (amino-acid sequence), a model predicts binding affinity. It thereby ranks a library without generating explicit poses. Deep learning has moved fast on this task [27], mostly benchmarked on the DAVIS kinase panel [28]. Approaches run from the sequence-convolutional DeepDTA [29] through self-attention-augmented convolutional models [30]. They extend to graph neural networks with self-attention pooling [31] and interaction transformers [32]. The spectrum also includes multimodal graph–sequence encoders [33,34] and graph-transformer early-fusion architectures [35]. Meanwhile, community benchmarks such as MoleculeNet have standardised these comparisons [36]. The second task is absorption, distribution, metabolism, excretion, and toxicity (ADMET) prediction. A tight binder is of little use if it is insoluble, poorly permeable, rapidly metabolised, or cardiotoxic. ML models now predict endpoints such as blood–brain-barrier permeability with high accuracy [37]. They also flag toxicity or side-effect risk early from molecular descriptors [38]. Such models are deployed through integrated ADMET platforms and in silico ADME models [39,40]. Furthermore, they benefit from learned molecular representations [41,42], and they guide formulation and delivery [43,44,45,46]. A reproducibility gap holds these advances back: datasets are re-curated in ad hoc ways, splits are often undisclosed, and metrics differ between studies. The Therapeutics Data Commons (TDC) was built to close it, aggregating openly licensed, ML-ready datasets behind a uniform interface with standardised splits and metrics [47].

Two shortcomings persist. Affinity is often optimised apart from developability [48]. Moreover, performance is often reported as a single random-split accuracy. Such a figure overstates prospective utility, and it says nothing about when to trust one prediction. This work therefore starts from the decision rather than from the score. Its central contribution is a confidence-gated affinity–ADMET docking-triage cascade (CADT). The cascade combines ensemble uncertainty [49,50,51], an applicability-domain check [52,53], and predicted developability. These signals together decide which compounds need docking. That decision, a learned gate in front of docking, is the role played by Deep Docking and active-learning virtual screening [16,17]. CADT fills it differently. It triages on predicted affinity, ADMET, and ensemble uncertainty rather than on docking scores. Moreover, it gates the decision by model confidence. Hence, the cascade defers to docking when its own prediction should not be trusted. Three further aims support this contribution. The first is a fully reproducible pipeline for affinity and ADMET prediction on openly licensed TDC data. The second is a set of transparent, fast, auditable baselines that more elaborate models should be asked to beat. These baselines are molecular fingerprints with gradient boosting and a compact convolutional network. The third is a clear reading of affinity performance under cold-target and cold-drug splits. Consequently, the cost of generalisation is stated rather than hidden in a random split. The pipeline is built to be re-pointed at the specific targets of an ongoing medicinal-chemistry programme with little effort.

## 2. Results

### 2.1. Drug–Target Affinity

**Figure 1:**
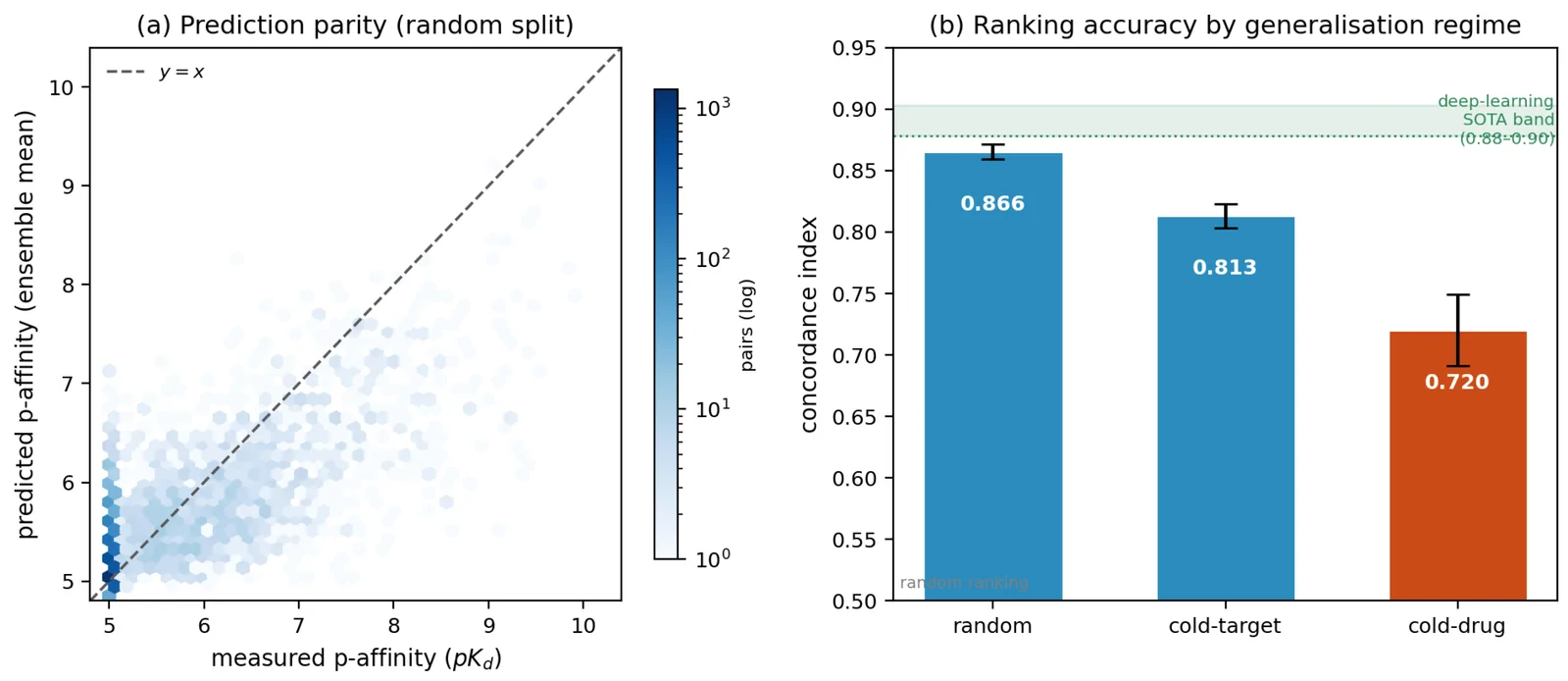
Affinity prediction on DAVIS. (**a**) Predicted versus measured p-affinity. (**b**) Concordance index by generalisation setting.

Affinity performance on DAVIS is summarised in Table 1 and Figure 1, averaged over five seeds. Under the random split, the fingerprint gradient-boosting baseline reached a concordance index of 0.866±0.006 and a Pearson correlation of 0.757±0.013. Withholding whole proteins (cold-target) lowered the CI to 0.813±0.010. Withholding whole drugs (cold-drug) lowered it further to 0.720±0.029 (Pearson 0.466). The wide interval reflects only 14 unique held-out drugs. The compact convolutional network, trained for 25 epochs on the same five seeds within a CPU budget, reached 0.830±0.011, below the baseline (non-overlapping 95% intervals). This is not an architectural verdict. A CPU-limited, untuned network is not the GPU-trained model of the original reports. Hence, it shows only that a small deep network does not beat the transparent baseline within this budget.

Table 2 lists recent deep-learning DTA models on the same DAVIS random split. These GPU-trained graph, transformer, and attention architectures reach concordance indices of roughly 0.88–0.90. On the same metric, the transparent baseline here (0.866) is level with the sequence-convolutional DeepDTA (0.878). However, it trails the best graph and transformer models by a few hundredths. The comparison is like-for-like in metric but not protocol (the cited models use their own splits and cross-validation), so it situates the baseline rather than ranking it. The contribution is therefore not an accuracy record but a reproducible, uncertainty-aware pipeline that is nonetheless competitive. It is fully inspectable and trained in about two minutes on one CPU. Moreover, it is paired with cold-split estimates and the triage cascade below.

Roughly 72% of DAVIS measurements sit at the 10,000 nM ($pK_{d}=5$) detection limit. Therefore, the concordance index is evaluated over the 48% of pairs whose labels differ. Much of the headline CI therefore reflects the easy separation of this censored bulk from the active minority. Accordingly, ranking among the uncensored pairs is a stricter test. Restricted to those pairs, the CI falls to 0.71 (random), 0.64 (cold-target), and 0.52 (cold-drug), with a higher MSE, since capped values are treated as exact. These figures are reported alongside the standard all-pairs CI, kept for comparability with the DTA literature. Triage is stable to the activity threshold: raising it from $pK_{d}\geq 6$ to ≥7 to ≥8 raises the enrichment factor monotonically (cold-target EF=3.6, 4.4, 5.0). Two censor-aware alternatives were also trained: an accelerated-failure-time model treating capped affinities as right-censored, and a variant trained only on uncensored pairs. Neither improved uncensored ranking (uncensored CI 0.69–0.70 for all three: standard 0.70, AFT 0.69, uncensored-only 0.70). This indicates that the ∼0.70 ceiling reflects the 2D fingerprint–composition representation, not the censoring loss.

To test transferability, the identical pipeline was applied to the larger KIBA benchmark [54]. The pipeline retained Morgan fingerprints, gradient boosting, and the same five seeds and evaluation. KIBA comprises 2068 compounds and 229 kinases, roughly thirty times as many unique drugs as DAVIS. Ranking accuracy held up, with a concordance index of 0.839±0.002 under the random split. The cold-target split gave 0.727±0.023, reproducing the DAVIS pattern on a chemically broader set. The baseline is therefore not an artefact of the small DAVIS drug space, though both benchmarks remain kinase-centric and a fully diverse target space is untested.

### 2.2. Representation Ablation

The uncensored figures above raise the question of whether the ranking ceiling is a property of the task or of the descriptors. Both halves of the pair representation were therefore replaced with stronger alternatives. The design was a full factorial over three drug representations and two target representations. Each cell used the same three splits and the same five seeds, giving 90 fits in total. Table 3 reports them as mean ± standard deviation over seeds. Two metrics are given. The first is the standard all-pairs concordance index. The second, ${\mathrm{CI}}_{\mathrm{unc}}$, is restricted to the uncensored pairs, which is what the ceiling question concerns. The cold-drug split is omitted from that table. With only 14 held-out drugs, its standard deviation reaches 0.064, and it separates no representation from any other. Its values are quoted in the text below instead.

On the drug side, Mordred did not displace the fingerprint. It gained at most 0.007 CI, within one to two standard deviations. Concatenating the two blocks added nothing beyond that, at roughly 1.7 times the feature count. The more useful difference appeared under drug-distribution shift. There the fingerprint proved markedly the steadier of the two. Across seeds on the cold-drug split its standard deviation was 0.023. The corresponding figures were 0.064 for Mordred and 0.061 for the concatenation. Continuous descriptors thus became least dependable exactly where a screening campaign most needs dependability. A compact binary fingerprint is therefore retained. It is kept not for want of an alternative, but because the alternative was measured and did not earn the swap.

On the target side, ESM-2 helped, modestly and where theory says it should. Under the cold-target split, it lifted the CI from 0.813 to 0.831. The uncensored CI rose from 0.653 to 0.668. That is the regime in which every test protein is unseen, so a richer sequence representation has most to offer. Under cold-drug, it changed nothing at all (0.720 against 0.717). This is the control one would hope for. No protein representation should rescue predictions when it is the drugs that are novel.

The ceiling itself, however, survived. Upgrading both halves at once raised the feature count from 1444 to 2650. That moved the uncensored CI from 0.701 to 0.721 on the random split, and from 0.653 to 0.669 on cold-target. A gain near 0.02 for so large an investment does not support that explanation. Two-dimensional fingerprints and composition descriptors are not what caps this task. The constraint lies elsewhere. It rests in the heavy label censoring described above, and in the absence of structural or pose information. This finding also justifies the transparent baseline retained throughout the rest of this work. Its ranking sits within a few hundredths of the best representation tested here, at a fraction of the cost.

### 2.3. External Validation on BindingDB

Both benchmarks used so far are kinase panels, and both are heavily censored. Two questions therefore remain open. The first is whether the pipeline transfers to a broader target space. The second is whether the ranking ceiling of the previous section belongs to the method or to DAVIS. The identical pipeline, unchanged in every setting, was applied to BindingDB $K_{d}$ [55]. That collection is larger and far more diverse. It holds 52,274 pairs over 10,661 compounds and 1413 protein targets. DAVIS, by comparison, holds 25,772 pairs over 68 compounds and 379 kinases. Only 343 BindingDB targets appear in the DAVIS panel, so roughly three-quarters of the target space is new. Median target length also rises from 379 to 465 residues. The collection is almost entirely uncensored, with 0.1% of affinities at a detection limit against 72% for DAVIS. Results are given in Table 4.

Three readings follow. The first settles the ceiling question raised in Section 2.2. On BindingDB, the uncensored concordance index reaches 0.836 under the random split. That is well above the 0.701 obtained on DAVIS with the same descriptors and the same model. A ceiling near 0.70 is therefore not a property of the descriptors at all. It is a property of DAVIS. Its informative pairs are the small uncensored minority left once two-thirds of the panel is pinned at a limit. This agrees with the representation ablation, which found that better descriptors bought almost nothing. Together, the two experiments identify the label distribution, not the feature space, as the true constraint.

The second reading is that the pipeline transfers. Its target space here is three-quarters outside the kinome, and its compound space is roughly 150 times larger. Ranking accuracy is nonetheless at least as good as on the kinase panel, once censoring is accounted for. The claim of this work is therefore not restricted to kinases. Its cascade results, reported below, still are.

The third reading concerns what a cold split actually measures. It is a caution rather than a result. The ordering of the two cold protocols reverses between the collections. On DAVIS, withholding drugs is much harder than withholding targets (0.615 against 0.653 uncensored). On BindingDB, the reverse holds (0.776 against 0.719). The explanation lies in the shape of each collection. DAVIS offers 379 closely related kinases but only 68 compounds. An unseen compound is therefore a long extrapolation, while an unseen kinase resembles many seen ones. BindingDB inverts both counts. The difficulty of a cold split is thus a statement about the dataset as much as about the model. Cold-split scores should not be compared across collections without that caveat. Absolute error also rises on BindingDB, with an MSE of 0.706 against 0.291. This is expected once the label range widens from four to eight log units. The ranking metrics are the comparable ones here.

### 2.4. ADMET Developability

**Figure 2:**
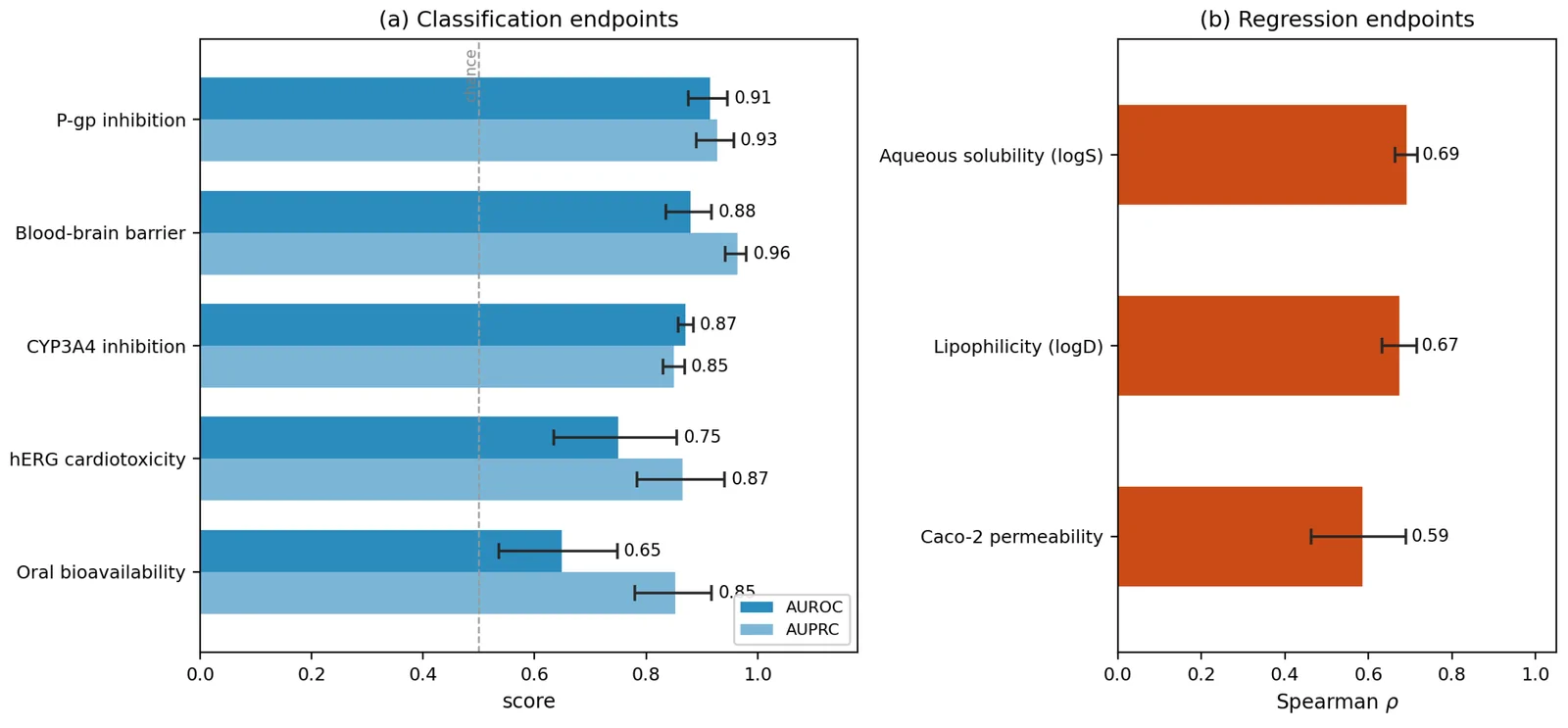
ADMET endpoint performance.

The ADMET panel (Table 5, Figure 2) used scaffold splits. Binary endpoints reached AUROC between 0.65 and 0.91. The strongest endpoints were P-glycoprotein inhibition (0.914) and blood–brain-barrier penetration (0.879). The latter is relevant to central-nervous-system programmes such as GSK3β [18]. It nevertheless sits below the 0.95 AUROC of a dedicated model trained on a much larger database [37]. CYP3A4 inhibition and hERG cardiotoxicity reached AUROC values of 0.870 and 0.749, respectively. Aqueous solubility and lipophilicity gave Spearman correlations of 0.691 and 0.675, respectively. Oral bioavailability was the hardest endpoint (0.649), consistent with its noise and small size. Bootstrap intervals expose the effect of test-set size. The large CYP3A4 set (n=2467) gives a tight interval ([0.857, 0.884]). By contrast, the small hERG and oral-bioavailability sets (n=132 and 128) span roughly 0.2 in AUROC. AUPRC must be read against prevalence. The blood–brain-barrier AUPRC of 0.96 partly reflects an 81% positive rate. By contrast, the balanced P-glycoprotein set gives a more informative 0.93. Of the eight endpoints, only the hERG and solubility models feed the CADT cascade. These are the liabilities most likely to disqualify a potent kinase inhibitor. The other six form a developability panel, and can be added without changing its structure. The hERG classifier is a caveat. It feeds the gate, yet rests on only 132 compounds (AUROC [0.635, 0.855]), so its calls are provisional. This is a further reason why developability is treated as a soft flag rather than a hard cut-off.

Those bootstrap intervals understate the problem, and it is worth being explicit about why. Resampling the test set holds the split itself fixed. It therefore captures sampling noise, but not the larger movement produced by redrawing the scaffold split. Every endpoint was refitted over five scaffold-split seeds, reported in the last column of Table 5. That spread is the honest uncertainty on each headline value. Three consequences follow.

First, the single-split figures quoted above for the two smallest endpoints sit low in their own seed distributions. Across seeds hERG averages 0.806±0.029 and oral bioavailability 0.707±0.049. The fixed split gave 0.749 and 0.649, respectively. Second, and unexpectedly, the least stable endpoint is neither of these. It is Caco-2 permeability, whose Spearman correlation ranges from 0.316 to 0.827 across the five seeds (0.686±0.211). A regression endpoint scored on 182 scaffold-split compounds does not resolve to three decimal places. It is set in bold in Table 5 for that reason. Third, the instability is largely, though not entirely, a question of size. Rescoring the well-behaved CYP3A4 model on subsamples of its own test set gives a 95% band of width 0.103 at n=128. The corresponding band for hERG at n=132 is 0.175 wide. Sample size therefore accounts for most of the spread, and the assays themselves account for the rest.

In summary, none of these endpoints should be read as a precise ranking. None is used as a hard filter anywhere in this work.

### 2.5. CADT Performance

**Figure 3:**
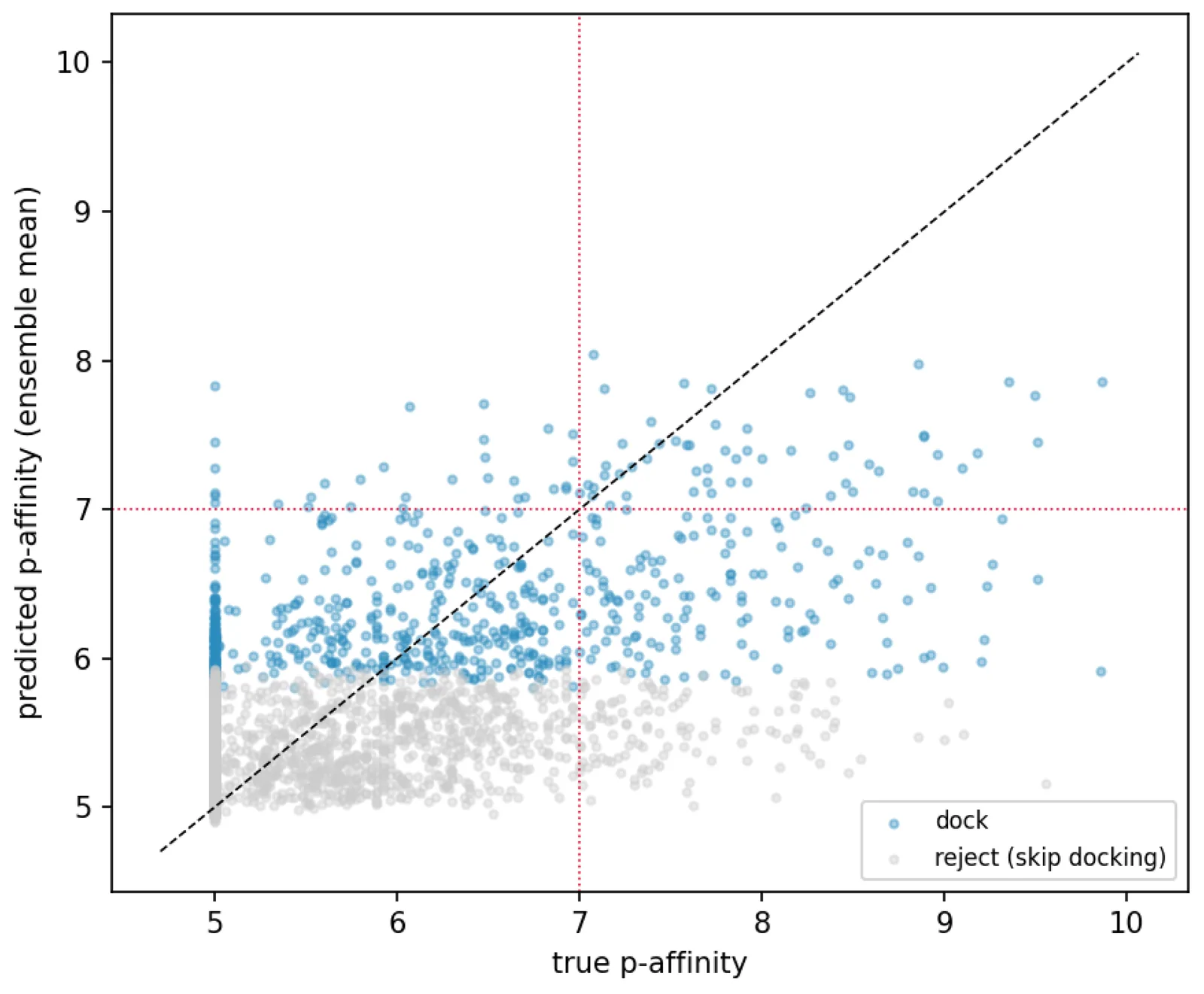
CADT routing on the DAVIS cold-target split. Blue pairs proceed to docking, grey pairs are skipped.

Table 6 and Figure 3 report the cascade on the cold-target split (5168 pairs, 318 true strong binders, a 6.2% active rate). Docking every pair defines the reference (EF=1). The affinity-confidence gate routed only 710 pairs to docking, a projected 86.3% reduction in load. Notably, this gate retained 61.0% of the true strong binders. Moreover, the hit rate rose from 6.2% to 27.3% (EF=4.44). Under the easier random split, the same gate retained 84.8% of the strong binders at an 85.2% load reduction (EF=5.72), so the cascade degrades gracefully. Applying the ADMET developability filter as a hard gate was counterproductive. It shrank the docking set to 102 pairs, yet it cut active retention to 15.1%. This drop occurs since many potent kinase inhibitors carry predicted hERG or solubility liabilities.

To attribute performance to individual components, Table 7 compares each signal at a matched docking budget. On the random and cold-target splits, the uncertainty term, the applicability-domain gate, and the soft-ADMET score behaved alike. All three were statistically indistinguishable from ranking by predicted affinity alone, with overlapping bootstrap intervals. Cold-target recall, for instance, was 0.610 for every variant. This equivalence arises since no test pair falls outside the applicability domain there. The components earn their place only under drug-distribution shift. On the cold-drug split, 64% of pairs are out-of-domain. Consequently, the applicability-domain gate refuses to confidently reject compounds the model is extrapolating on, forcing them to docking. This is deliberately conservative: it lowers enrichment relative to naive affinity ranking (recall 0.65, EF=0.95 versus 0.84, EF=1.22) rather than improving it. The cascade is therefore best understood not as an accuracy gain but as an abstention mechanism. It costs nothing when the model interpolates. Moreover, it defers to docking when the model does not.

**Figure 4:**
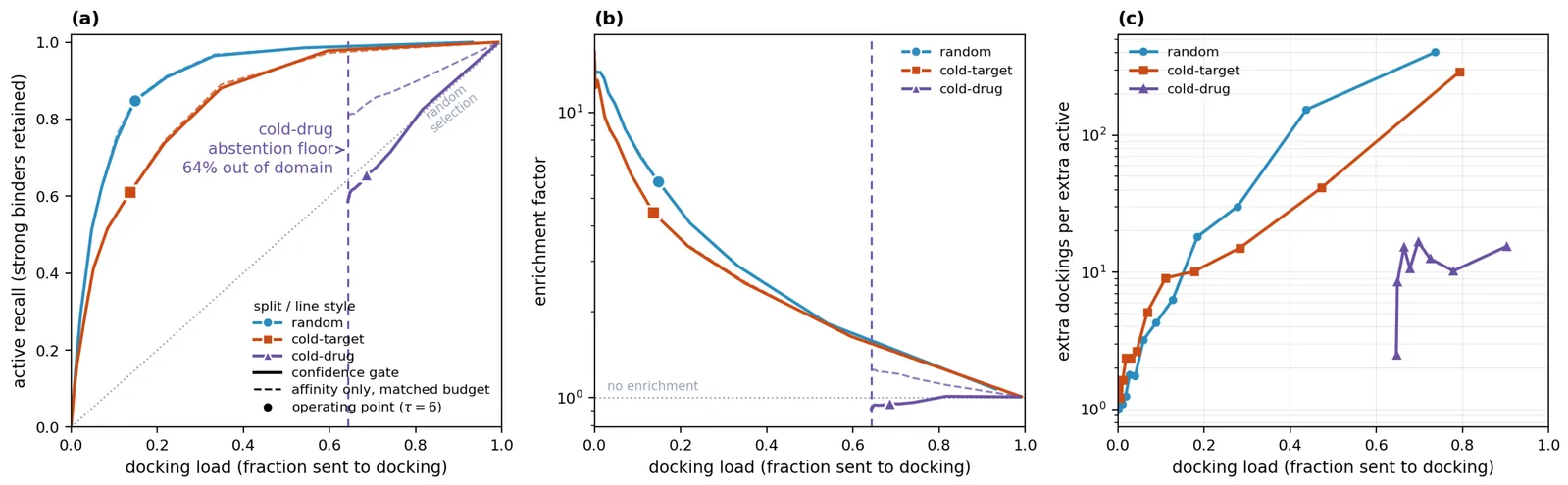
Docking load against recall, enrichment, and marginal cost. (**a**) Active recall, (**b**) Enrichment factor, and (**c**) Additional dockings per additional active. Solid curves are the confidence gate, whereas dashed curves are affinity-only ranking at the same budget. Symbols mark the operating point at τ = 6.

A single operating point, however, tells a screening chemist little about the choice they actually face. The rejection threshold τ was therefore swept across its full range on all three splits. At every achieved docking load, the gate was compared against plain affinity ranking at the same budget. Table 8 and Figure 4 report the result. Throughout, κ=1, and load denotes the fraction of pairs sent to docking. The row at τ=6 is the operating point already reported in Table 6. In Figure 4, the solid curves are the gate and the dashed curves affinity ranking. Three things emerge, and the first was not anticipated.

First, the gate has an abstention floor, and on cold-drug that floor is high. An out-of-domain pair is never cheaply rejected. The smallest docking load the cascade can reach is therefore exactly the out-of-domain fraction. On cold-drug, that is 3411 of 5306 pairs, so the load cannot fall below 64.3%. Raising τ from 6.5 to 9.0 moves it by barely one percentage point. Under genuine drug-distribution shift, the cascade therefore cannot deliver large savings at all. The 86% reduction obtained on cold-target should not be read as transferable to that regime.

Second, the comparison against affinity ranking depends on the regime, and the direction is not flattering everywhere. On the random and cold-target splits, the two are indistinguishable at every operating point. Recall differs by at most 0.012. On cold-drug, the gate is worse on both axes at every matched budget. It gives up 0.15 to 0.20 of recall and 0.2 to 0.3 of enrichment. This is the price of abstention, stated plainly. The gate spends budget on out-of-domain pairs that ranking would have skipped. Against retrospective labels that spending looks wasteful. Its justification is not visible in these metrics. The uncensored concordance index on cold-drug is only 0.615, so the model’s confident rejections there are the least trustworthy.

Third, the marginal cost of a looser gate is the quantity worth planning against. It varies over two orders of magnitude. On the random split, widening the load from 14.8% to 22.2% costs about 18 extra dockings per extra active. From 33% to 54%, the price rises to 153, and from 54% to 93%, to 402. A campaign can therefore read its own stopping point off this curve, given the cost of one docking run. Cold-drug behaves quite differently. Across its whole reachable range, the marginal cost stays flat between roughly 10 and 17, with no knee to aim for. That is another way of seeing that the ranking carries little signal in that regime.

In summary, the sweep establishes three points. The gate cannot save much under drug shift. It costs recall there rather than adding it. Its operating point should be chosen from the marginal-cost curve.

### 2.6. Robustness, Uncertainty Calibration, and Triage Analysis

**Figure 5:**
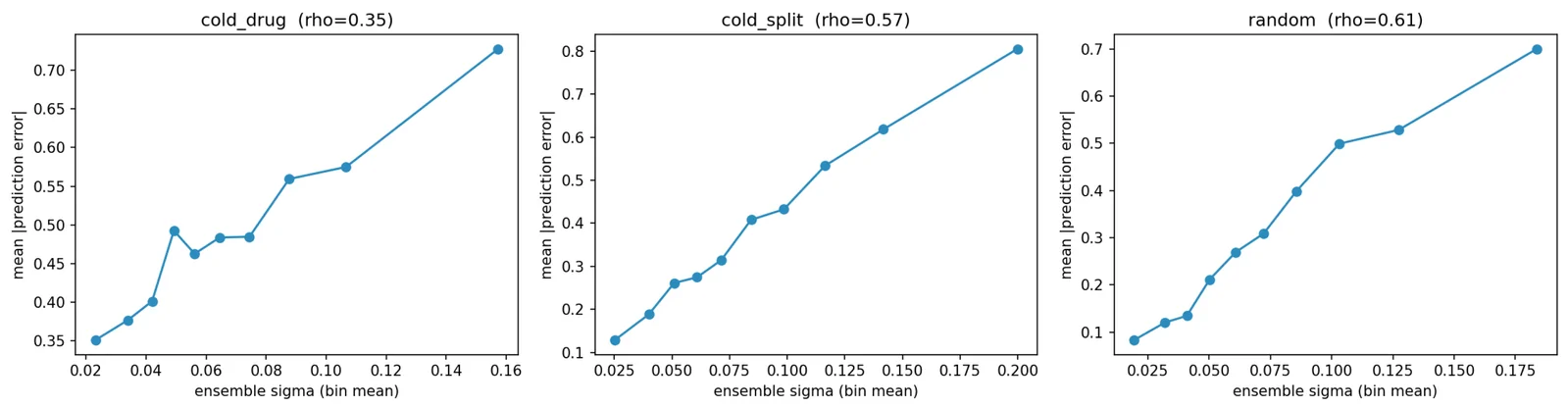
Uncertainty calibration: mean absolute error against ensemble standard deviation, by decile.

**Figure 6:**
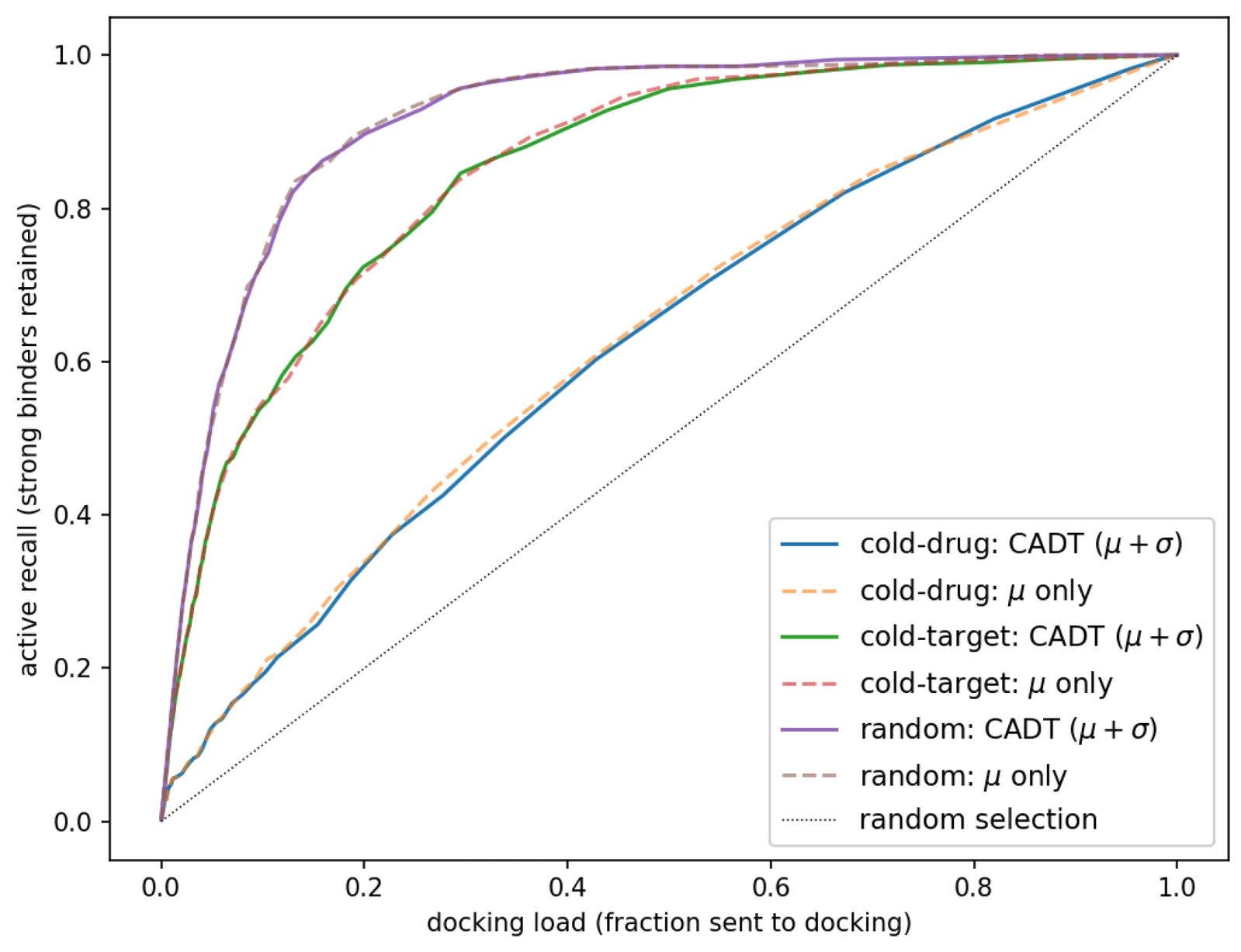
Triage operating curve: active recall against docking load for the three splits.

First, the ensemble uncertainty is well calibrated. The Spearman correlation between σ and absolute error is positive in every regime (0.61 random, 0.57 cold-target, 0.35 cold-drug). Moreover, mean error rises monotonically across σ deciles (Figure 5). Hence, the CADT abstention signal is well founded, even though on homogeneous DAVIS, the gate coincided with plain score ranking. The applicability-domain check activates where expected: on cold-drug, it flagged 64% of pairs as out-of-domain, versus 0% on random and cold-target. Second, a fixed docking budget spent by affinity, or by a soft affinity–developability score, retained roughly twice as many actives as a hard ADMET filter. This held in every regime (Table 9). Notably, on cold-drug, the hard filter even de-enriched the shortlist (EF 0.54). Therefore, developability is better used to re-rank a shortlist than to eliminate compounds. Finally, kinases with more strong binders were harder to predict (Spearman 0.61 between active count and mean error). In addition, the triage operating curve (Figure 6) confirms enrichment above the random diagonal.

Docking turns the projected saving into a measured one, and it also places the cascade beside the method it feeds. Table 10 reports both. Docking all 407 compound-target pairs consumed 99.8 hours of processor time. Docking only the pairs the gate retained consumed 15.0 hours. The measured saving of 85.0% confirms the 86.3% projected from compound counts in Section 2.5. Compounds the gate keeps are slightly more expensive to dock than those it rejects, which accounts for the small shortfall.

At an equal budget, the cheap gate selects better than the expensive method. Of the 407 pairs, 61 are true binders, a base rate of 15.0%. The gate routes 56 pairs to docking and 27 of them are true binders, a hit rate of 48.2% and an enrichment of 3.2. Selecting the 56 best-scoring pairs by docking instead recovers 11 true binders, a hit rate of 19.6% and an enrichment of 1.3. Over all 407 pairs, the predicted affinity tracks the measured value at ρ=0.55, while the docking score reaches ρ=0.13. On this panel, a pre-screen costing seconds therefore prioritises compounds better than the structure-based calculation it precedes.

Two qualifications follow from the same experiment. The first is that the docking score does not separate the retained pairs from the rejected ones. That is a statement about the scoring function rather than about the gate. The two sets differ sharply by measured affinity, with median $pK_{d}$ of 6.90 against 5.00. The second is that abstention is not free. Among the rejected compounds, 28 bind at least one of the six kinases and fail against at least one other. Their docking rank is better on the kinases they bind than on those they do not, by a paired test (p=0.005). Restricting this to the four receptors that meet a 2 Å redocking criterion leaves it in place (p=0.028).

## 3. Discussion

### 3.1. Principal Findings

Three points stand out from the present study. The first is that the CADT cascade renders the affinity–developability triage explicit and auditable. In the retrospective simulation, only 14% of the cold-target library is routed to docking, while 61% of the strong binders are retained. Furthermore, the ablation reported in Table 7 clarifies when each component of the cascade matters. When the screening library shares chemotypes with the training set, a simple gradient-boosted affinity threshold is already sufficient. This is precisely the cold-target case emphasised here. Accordingly, the uncertainty and applicability-domain components neither help nor hurt in that regime. It is worth mentioning that a practitioner screening known chemotypes against a novel target should therefore adopt the bare affinity gate. The extra machinery may be skipped. However, the added components earn their place only with genuinely novel chemotypes, as in the cold-drug setting. There, the applicability-domain gate refuses to confidently reject compounds on which the model is extrapolating. Such pairs are therefore deferred to docking. Consequently, the recommendation flips and the full cascade is warranted, since abstention is worth its cost in enrichment. This graded guidance is more useful than a single monolithic pipeline. A separate design lesson also follows. A hard ADMET filter discards potent binders, whereas a soft affinity–developability score does not. Hence, developability belongs in a screening cascade as a re-ranking flag, not as a hard cut-off.

The second point is that transparent baselines are already useful in this setting. A Morgan fingerprint combined with gradient boosting is fully inspectable, and it trains in about two minutes on a single CPU. Moreover, it ranks DAVIS kinase pairs with a concordance index near 0.87. On the same metric, this matches the sequence-convolutional DeepDTA (0.878). It sits a few hundredths below the 0.88–0.90 reached by the strongest GPU-trained graph and transformer models [29,30,31,33,34,35]. Each of those figures was obtained on its own split. Such performance is good enough as a cheap first pass in front of docking. In addition, its simplicity makes it easy to audit. It is worth noting that the same interpretable gradient-boosting recipe is now widely used for pharmaceutical property prediction [37,43]. Furthermore, model transparency is itself an active concern in AI-driven drug discovery [7]. Meanwhile, the compact convolutional network did not beat the fingerprint baseline within the imposed CPU budget. This outcome runs against data-rich sensor, imaging, and simulation settings, where deep models clearly outperform classical pipelines [56,57,58,59,60]. Therefore, for the affinity pre-screen intended here, a transparent model is preferable. This holds unless a large deep-learning training budget is available.

The third point concerns the gap between random and cold splits, which matters most for real use. A model that appears strong under a random split may be exploiting the reappearance of familiar proteins. Transferable chemistry is not necessarily being learned. The drop from a CI of 0.866 under the random split to 0.813 for cold-target and 0.720 for cold-drug makes this concrete. Therefore, for prospective screening against a novel target or chemotype, the cold-split numbers are the honest expectation. Notably, multi-target computational pipelines are now standard in oncology and neuroscience drug discovery [8]. Within such pipelines, honest generalisation estimates of this kind are essential.

The reported reduction in docking load was originally presented as a projection. It has now been measured, in Section 2.6, at 85.0% against the 86.3% projected. Nevertheless, a prospective study on a live medicinal-chemistry programme remains the natural next validation. Two extensions follow from the limitations set out below. The first is a geometry-aware domain criterion, which is a priority. Such a criterion could be built on a learned molecular encoder or on docking-pose features. Moreover, the released pipeline is structured so that a learned encoder or an additional target can be substituted. The surrounding evaluation need not be rewritten. A second is the coupling of the pre-screen to the author’s structure-based workflows, which is a concrete next step. Relevant targets include GSK3β, BRPF1, and EGFR [18,19,20].

### 3.2. Limitations

It is worth noting that the limitations of the present study are gathered in one place here. They are therefore not left scattered through the discussion. Four areas are concerned, namely, the evaluation data, the models, the cascade, and the docking test.

Both affinity benchmarks used for the cascade are kinase panels, so the cascade results are kinase results. The broader BindingDB validation of Section 2.3 covers the affinity models alone. Furthermore, DAVIS is heavily censored, and that censoring rather than the descriptors sets the apparent ranking ceiling. On the developability side, two ADMET endpoints rest on fewer than 140 test compounds. Caco-2 permeability is also unstable across scaffold splits. The molecular representation is two-dimensional throughout. Accordingly, the applicability-domain gate measures topological rather than structural proximity. A compound judged in-domain may therefore still bind in a pose the model cannot anticipate. Moreover, the binary in-domain call of the gate is a coarse proxy for the true extrapolation risk. It should also be noted that the ADME-T endpoints are modelled independently of one another. Hyperparameters were fixed in advance rather than tuned, which removes selection bias at the cost of accuracy. The gate also has a structural floor, since it never cheaply rejects an out-of-domain pair. Consequently, under drug-distribution shift, the smallest load it can reach equals the out-of-domain fraction. That fraction is 64.3% on the cold-drug split.

Five further qualifications attach to the docking measurement of Section 2.6. First, the six kinases were chosen as those holding the most rejected true binders. The subset is therefore the hardest slice of the split, rather than a representative one. Active recall on it is 44%, against the 61% obtained across all 76 cold-target kinases. Second, two of the six receptors, LOK and TAOK3, redock their own ligand to 2.7 and 2.5 Å. Both values therefore fall short of the usual 2 Å criterion. Third, receptors were held rigid, one structure was used per target, and one scoring function was applied. Accordingly, the comparison with docking is specific to this protocol. Fourth, the measured affinities were known in advance. The exercise is therefore retrospective rather than blind. Fifth, the ligands were docked in their neutral forms, since ionisation was inherited from the source SMILES rather than assigned at pH 7.4. Forty of the 68 compounds carry an aliphatic amine that would be protonated at physiological pH. Their electrostatic complementarity with the receptor is therefore approximated. The receptor structures were likewise used as deposited, without energy minimisation.

## 4. Materials and Methods

**Figure 7:**
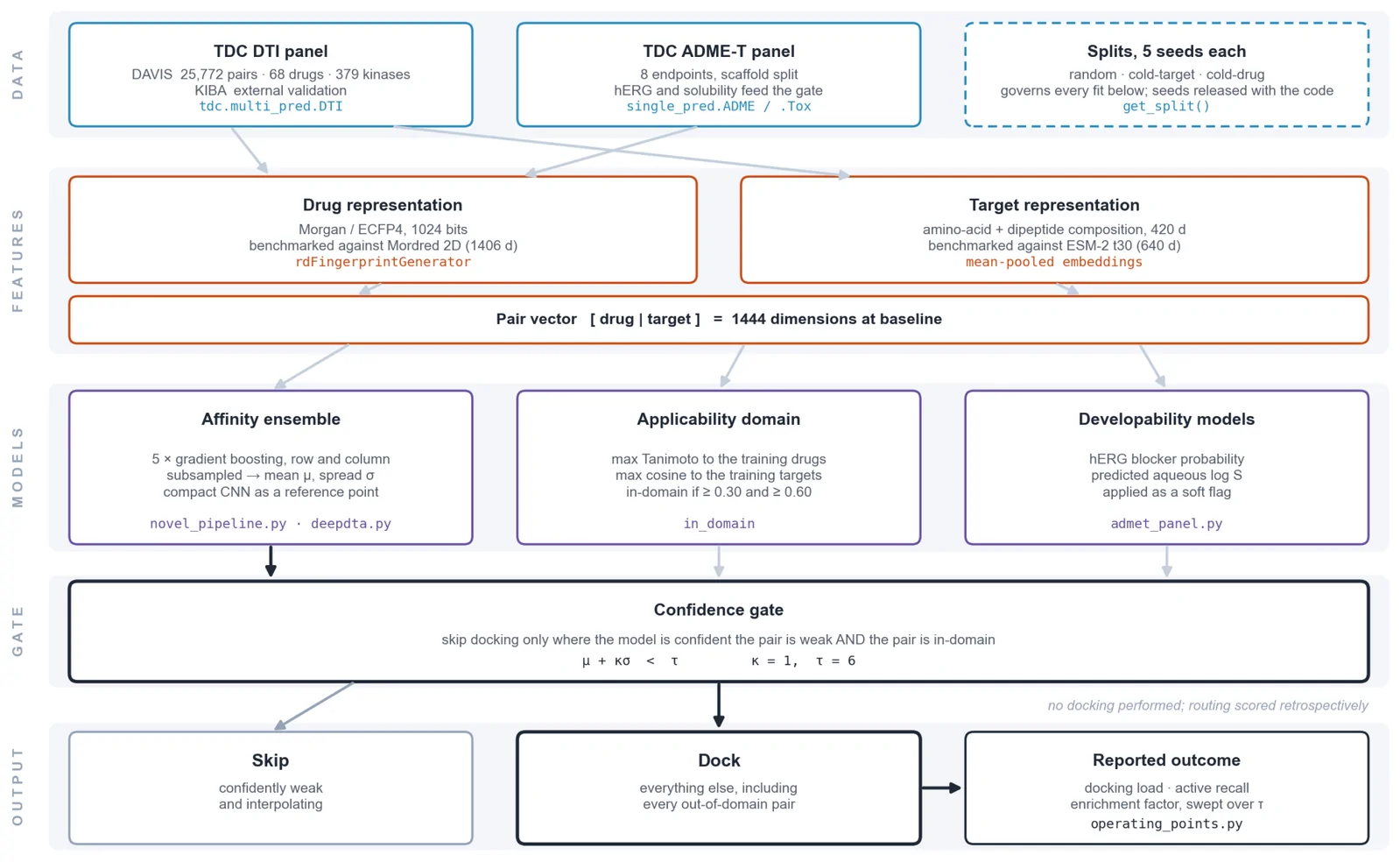
The CADT pipeline, from open data to routing decision.

The complete pipeline, as it is actually executed, is set out in Figure 7. Data are drawn from openly licensed Therapeutics Data Commons (TDC) collections. Each entry is then reduced to a single pair vector. That vector feeds three components, whose outputs meet at the confidence gate. Only pairs that the affinity ensemble judges weak with confidence are withheld from docking. Moreover, such pairs must additionally lie inside the applicability domain. Each band of the figure corresponds to one stage. Furthermore, every box names the script or setting that produces it. The account given here can therefore be read directly against the released code. It is worth noting that the routing decision itself is scored retrospectively against the measured affinities. Its cost is separately measured by docking, as described in Section 4.5.

### 4.1. Datasets

All data used in the present study were obtained from the TDC platform (https://tdcommons.ai, accessed on 19 July 2026) [47]. Notably, this platform provides openly licensed, ML-ready datasets together with standardised splits. Affinity modelling was based on the DAVIS kinase panel [28]. This panel contains 25,772 pairs, which were formed from 68 kinase inhibitors and 379 kinase proteins. Dissociation constants ($K_{d}$) were reported on the $pK_{d}$ scale. Developability modelling, in turn, drew on eight ADMET datasets. These endpoints were aqueous solubility, lipophilicity, Caco-2 permeability, blood–brain-barrier penetration, P-glycoprotein inhibition, oral bioavailability, CYP3A4 inhibition, and hERG cardiotoxicity.

### 4.2. Molecular Representation

Drugs were represented by 1024-bit Morgan (extended-connectivity) fingerprints of radius 2, which were computed with RDKit (https://www.rdkit.org). This representation follows the recommendations of the open benchmarking platform of Riniker and Landrum [61]. Proteins were, in turn, represented by a 420-dimensional descriptor. That descriptor concatenates amino-acid (20-dim) and dipeptide (400-dim) composition. Each descriptor was computed once and then reused across pairs.

Neither default representation was adopted on faith. Both were instead tested against a stronger alternative in the ablation of Section 2.2. That check was warranted, since a ranking ceiling may reflect the descriptors rather than the task itself. On the drug side, the alternative was the Mordred two-dimensional descriptor block [62]. Of its 1613 descriptors, those that were numeric, finite, and non-constant were retained, which left 1406. That filter was fitted on the training drugs alone and then applied unchanged to the test drugs. Consequently, no test molecule influenced which descriptors existed. Values were furthermore clipped to the training range. An unseen molecule therefore cannot inject an outlier that the model has no basis to handle. On the protein side, the alternative was ESM-2 [63], a protein language model, taken in its 150M-parameter form. Each sequence was embedded once and then mean-pooled over residues to 640 dimensions. Every combination was run over the same five seeds and the same three splits as the baseline.

### 4.3. Models

Two model families were evaluated for affinity prediction. The first was gradient-boosted regression trees [64], in the XGBoost implementation. This model was applied to the concatenated drug–protein descriptor. Notably, gradient boosting has a strong track record across heterogeneous prediction tasks, including magnetic-levitation design [65]. The second was a compact DeepDTA-style convolutional network [29].

This subsection specifies the convolutional network in full. The specification covers the input encoding, the two convolutional branches, the dense head, and the training schedule. It is worth noting that this detail is given deliberately, since the reported result is otherwise not reproducible.

The network inputs were label-encoded at character level rather than pretrained. Two alphabets were used, namely, 63 SMILES symbols and the 22 amino-acid codes. Each alphabet reserved one further index for padding. SMILES strings were truncated or padded to 100 characters, whereas protein sequences were truncated or padded to 1000 residues. Both alphabets were then mapped through learned 128-dimensional embedding tables. Meanwhile, the padding index was held at zero in both tables.

The drug branch applied three one-dimensional convolutions of 32, 64, and 96 filters, at kernel widths 4, 6, and 8. The protein branch used the same filter counts with wider kernels of 4, 8, and 12, matching the longer sequence. A rectified linear unit followed each convolution. Each branch was then max-pooled over positions to a single 96-dimensional vector. The two vectors were concatenated and passed to a dense head of 1024, 512, and 1 units. A rectified linear unit and 0.1 dropout followed the first two layers. In total, the network held 918,785 trainable parameters.

Training minimised the mean squared error with Adam, at a learning rate of $10^{-3}$ and a batch size of 256. The schedule ran for at most 25 epochs. Training was stopped early if the validation error failed to improve for 10 epochs. The best validation weights were then restored. All runs were performed on CPU and took roughly one hour per seed. Furthermore, they used the same five seeds and the same random split as the gradient-boosting baseline.

For ADMET, gradient-boosted trees were used throughout. Each endpoint was modelled as a classifier or as a regressor, according to the endpoint type.

### 4.4. Data Splits and Evaluation

Three affinity protocols were employed in the present study: random (interpolation), cold-target (no test protein in training), and cold-drug (no test drug in training). Meanwhile, the ADMET endpoints were evaluated under scaffold splits. Affinity was assessed by mean squared error (MSE), root mean squared error (RMSE), Pearson and Spearman correlation, and the concordance index (CI). It is worth noting that the CI is the standard DTA ranking metric [66,67]. Accordingly, it was computed as in the literature benchmarked against [29,31]. Over all test pairs with differing affinities, the CI is the fraction whose predicted order is correct, with ties counting one half. This distinction matters, since roughly two-thirds of DAVIS affinities are censored at the 10,000 nM ($pK_{d}=5$) limit. Therefore, this comparable-pair definition is the appropriate metric. A rescaled Kendall’s τ would instead penalise those ties. Consequently, it would understate ranking by about 0.10. The random and cold-target protocols were repeated over five seeds, reported as mean ± 95% confidence interval. In addition, bootstrap 95% intervals were computed on the test predictions [68]. Binary ADMET endpoints were scored by the area under the ROC curve (AUROC), the area under the precision–recall curve (AUPRC), and accuracy. Similarly, continuous endpoints were scored by the mean absolute error (MAE), RMSE, and Spearman correlation. Split sizes are reported in Table 11. Hyperparameters were fixed a priori and kept identical across all splits and seeds (gradient boosting: 600 trees, depth 8, learning rate 0.05, subsampling 0.8). Hence, this design precludes selection bias. Moreover, all three protocols share the same five seeds (1–5), so that within-protocol comparisons are paired.

### 4.5. Docking Validation

Furthermore, the abstention of the cascade was also examined against docking itself. Six kinases were drawn from the cold-target test split of seed 42, so the affinity model had never seen them. First, the cold-target kinases holding the most rejected true binders were identified. Second, only those possessing a crystal structure with a drug-like co-crystal ligand were retained. The resulting targets are VEGFR2 (PDB 4ASD), BRAF V600E (3OG7), GSK3β (6Y9S), LOK (6EIM), AURKC (6GR8), and TAOK3 (6BDN). All 68 DAVIS compounds were then docked into each target with AutoDock Vina 1.2.7 [69,70]. An exhaustiveness of 32 and a fixed seed were used throughout. Receptors retained chain A alone and were held rigid, whereas all waters were removed. Furthermore, every box was validated by rebuilding its own co-crystal ligand from SMILES and redocking it. It is worth noting that sorafenib is the co-crystal ligand of 4ASD. Accordingly, it is omitted from the ranking of that target to avoid circularity. Run times were recorded under identical conditions. Hence, the ratio between subsets remains meaningful, although the absolute times reflect concurrent execution.

Ligand and receptor preparation followed a fixed procedure. Each ligand was built from its parent SMILES after salt stripping. Hydrogens were added, and a single conformer was embedded with ETKDGv3 under a fixed random seed. That conformer was then energy-minimised with the MMFF94 force field, for at most 2000 iterations. Meeko 0.7.1 wrote the resulting PDBQT file, merging non-polar hydrogens and assigning rotatable bonds. It should be stated that ligand ionisation was inherited from the source SMILES rather than assigned at physiological pH. All 68 SMILES are formally neutral, so every compound entered the search in its neutral form. Receptors were reduced to chain A, and all waters and heteroatoms were deleted. Polar hydrogens were then added, non-polar hydrogens were merged, and Gasteiger charges with AutoDock atom types were assigned. Histidine residues were kept in the standard neutral form, without pH-dependent tautomer assignment. The receptor coordinates were used as deposited, without energy minimisation, and were held rigid throughout. Accordingly, no induced fit was modelled.

### 4.6. The CADT Cascade

Beyond scoring, a cascade decides which compounds require docking, and three signals are combined per pair. The first signal is Affinity with uncertainty. An ensemble of five gradient-boosted regressors with row and column subsampling yields a predicted p-affinity μ and an epistemic uncertainty σ. A second signal is the Applicability domain, a gate that compares each pair against the training set. A pair is in-domain only if the drug’s maximum Tanimoto similarity and the target’s maximum cosine similarity exceed preset floors of 0.30 and 0.60, respectively. A third signal is Developability, since independent TDC hERG and solubility models flag liabilities on the drug. Notably, a pair is rejected without docking only when two conditions hold together. The first condition requires the model to be confident that the pair is a weak binder (μ+κσ<τ, with κ=1 and τ=6). The second condition requires the pair to be in-domain. All other pairs proceed to docking, whereas developability is applied as an optional secondary filter. Actives were defined as pairs with $pK_{d}\geq 7$ ($K_{d}\leq 100$ nM). CADT was evaluated primarily on the cold-target split, which models prospective screening against novel targets. It is important to note that no docking was performed. The cascade was instead evaluated retrospectively against each pair’s measured affinity, so reported reductions in docking load are projections rather than measured savings. To attribute performance, the cascade was compared against four baselines at a matched docking budget. These baselines were ranking by affinity alone, affinity ± uncertainty, the applicability-domain gate, and a soft affinity–developability score $s=\mu -\frac{1}{2}\;\left(\;p_{\mathrm{hERG}}+\;\max\left(0,\;-6-\log S\right)\;\right)$. Performance is reported as active recall, defined as the fraction of true actives reaching docking. Furthermore, the enrichment factor (EF) is also reported, namely, the hit rate among docked pairs relative to docking everything. Both metrics are accompanied by bootstrap 95% intervals.

### 4.7. Statistical Analysis

All statistical analysis was performed in Python 3.12.3, with SciPy 1.17.1, NumPy 1.26.4, and pandas 2.3.3. Monotonic association is reported as the Spearman rank correlation coefficient ρ, computed with scipy.stats.spearmanr. Linear association is reported as the Pearson coefficient, computed with scipy.stats.pearsonr. Confidence intervals on single-split metrics were obtained by a non-parametric percentile bootstrap. One thousand resamples of the test set were drawn with replacement, and the 2.5th and 97.5th percentiles are reported. Multi-seed values are instead given as the mean over five seeds, with a Student *t* interval at 95% confidence on n−1 degrees of freedom.

The paired comparison of docking ranks in Section 2.6 used the one-sided Wilcoxon signed-rank test, through scipy.stats.wilcoxon. Each compound is paired with itself, so that molecular size and shape cancel rather than being adjusted away. The two paired values are its mean docking rank on the kinases it binds and its mean rank on the kinases it does not bind. Twenty-eight compounds satisfy both conditions and enter the test over all six receptors, and seventeen enter the test over the four receptors that meet the redocking criterion. The same contrast was repeated as a sign-flip permutation test over 20,000 resamples, which agreed closely with the Wilcoxon result (p=0.004 and p=0.026, respectively). Those two versions of the contrast are reported without multiplicity correction. All other *p*-values are two-sided, and a threshold of α=0.05 was applied throughout.

## 5. Conclusions

The central result of the present study is CADT, a confidence-gated cascade that decides which compounds are worth docking. Docking six withheld kinases confirmed the projected saving as a measured one. Moreover, the gate proved the better prioritiser of the two. In a retrospective simulation, the cascade would route only 14% of a cold-target library to docking. Meanwhile, it would keep 61% of the true strong binders. An ablation showed that this triage is driven mainly by predicted affinity. The uncertainty and applicability-domain signals act as an abstention mechanism under distribution shift, rather than as an accuracy gain. Furthermore, the ablation confirmed that developability is best applied as a soft flag rather than a hard filter. The cascade rests on a compact and openly reproducible pipeline assembled on Therapeutics Data Commons data. This pipeline brings ML into a structure-based workflow at two points, namely, drug–target binding affinity and ADMET developability. Transparent, fast and auditable models gave solid baselines for both tasks. Notably, a CI near 0.87 was reached for kinase affinity and an AUROC up to 0.91 for developability endpoints. Meanwhile, multi-seed confidence intervals together with cold-target and cold-drug evaluations made the cost of generalisation explicit.

Three findings generalise beyond this dataset. The first is that a transparent baseline proved competitive with published deep-learning models, at a fraction of the computational cost. This comparison used the standard CI reported across the DTA literature. Accordingly, such a result argues for setting up these baselines before more elaborate architectures are adopted. A second finding is that honest evaluation changes the picture. Cold-target and cold-drug splits, multi-seed intervals and an uncensored-pair analysis together show that a single random-split score overstates prospective performance. Furthermore, ranking of the informative and uncensored compounds (CI≈0.70) is weaker than the headline value. A third finding is that ensemble uncertainty and applicability-domain checks are most useful as abstention mechanisms rather than as accuracy boosters. On chemically homogeneous data they add little. However, under drug-distribution shift, they flag extrapolation and defer the decision to docking. Consequently, these lessons apply broadly to ML pre-screens for compound prioritisation.

Several extensions follow from the present study. The docking-triage result is a retrospective projection. Therefore, a prospective docking study would turn projected savings into measured ones. The ∼0.70 ceiling on uncensored ranking points to richer molecular and target representations, such as learned graph encoders or protein language-model embeddings. Paired with censor-aware training objectives, these representations are the likely route to genuine accuracy gains. Furthermore, validation beyond the kinase-centric DAVIS and KIBA panels would further test transferability. In addition, coupling the pre-screen to ongoing structure-based programmes on targets such as GSK3β, BRPF1, and EGFR is a concrete next step. A reproducible, uncertainty-aware and honestly evaluated pipeline of this kind can cut the number of compounds that reach expensive structural simulation. Notably, it can do so without discarding the physical rigour that makes those methods valuable.

## Figures and Tables

**Table 1:** Drug–target binding-affinity prediction on the DAVIS kinase panel.

Model	Split	CI ↑	Pearson ↑	RMSE ↓
Morgan + gradient boosting	random	0.866±0.006	0.757±0.013	0.539±0.014
Morgan + gradient boosting	cold-target	0.813±0.010	0.627±0.033	0.650±0.058
Morgan + gradient boosting	cold-drug	0.720±0.029	0.466±0.080	0.798±0.103
Convolutional network	random	0.830±0.011	0.670±0.030	0.612±0.025

↑ higher is better; ↓ lower is better.

**Table 2:** Recent deep-learning DTA models on the DAVIS benchmark (best model per paper, random split), for context. Values are as reported in the cited papers.

Model	Approach	Ref.	MSE ↓	CI ↑
DeepDTA	sequence CNN	[29]	0.261	0.878
CSatDTA	CNN + self-attention	[30]	0.241	0.892
EMPDTA	multimodal + pocket detection	[34]	0.218	0.891
GEFormerDTA	graph-transformer early fusion	[35]	0.212	0.895
GRA-DTA	GraphSAGE + attention BiGRU	[33]	0.225	0.897
SAG-DTA	self-attention graph pooling	[31]	0.209	0.903
This work (Morgan + gradient boosting, random)			0.291	0.866

↑ higher is better; ↓ lower is better.

**Table 3:** Representation ablation on DAVIS.

Drug	Target	Dimension	Random		Cold-Target	
			CI	${\mathrm{CI}}_{\mathrm{unc}}$	CI	${\mathrm{CI}}_{\mathrm{unc}}$
Morgan (this work)	composition	1444	0.866±0.005	0.701±0.009	0.813±0.008	0.653±0.013
Mordred	composition	1406	0.871±0.004	0.708±0.008	0.815±0.009	0.653±0.014
Morgan + Mordred	composition	2430	0.873±0.004	0.709±0.009	0.816±0.008	0.655±0.013
Morgan	ESM-2	1664	0.874±0.005	0.714±0.010	0.831±0.006	0.668±0.015
Mordred	ESM-2	1626	0.878±0.005	0.720±0.007	0.830±0.009	0.672±0.016
Morgan + Mordred	ESM-2	2650	0.879±0.006	0.721±0.009	0.831±0.010	0.669±0.016

**Table 4:** The same pipeline on DAVIS and on BindingDB $K_{d}$.

Split	DAVIS (72% Censored)			BindingDB $K_{d}$ (0.1% Censored)		
	CI	${\mathrm{CI}}_{\mathrm{unc}}$	MSE	CI	${\mathrm{CI}}_{\mathrm{unc}}$	MSE
Random	0.866±0.005	0.701±0.009	0.291	0.836±0.001	0.836±0.002	0.706
Cold-target	0.813±0.008	0.653±0.013	0.424	0.719±0.033	0.719±0.033	1.275
Cold-drug	0.720±0.023	0.615±0.020	0.643	0.776±0.020	0.776±0.020	0.914

**Table 5:** ADMET endpoint prediction (Morgan + gradient boosting, scaffold split).

Endpoint	TDC Dataset	Type	$n_{\mathrm{test}}$	Prev.	Primary Metric [95% CI]	5 Splits
Blood–brain barrier	BBB_Martins	clf	406	0.81	AUROC 0.879 [0.836, 0.918]	0.905±0.025
P-glycoprotein inhib.	Pgp_Broccatelli	clf	245	0.51	AUROC 0.914 [0.875, 0.946]	0.901±0.024
CYP3A4 inhibition	CYP3A4_Veith	clf	2467	0.44	AUROC 0.870 [0.857, 0.884]	0.888±0.014
hERG cardiotoxicity	hERG	clf	132	0.73	AUROC 0.749 [0.635, 0.855]	0.806±0.029
Oral bioavailability	Bioavailability_Ma	clf	128	0.76	AUROC 0.649 [0.536, 0.748]	0.707±0.049
Aqueous solubility	Solubility_AqSolDB	reg	1997	–	Spearman 0.691 [0.664, 0.717]	0.717±0.031
Lipophilicity	Lipophilicity_AstraZeneca	reg	840	–	Spearman 0.675 [0.632, 0.715]	0.683±0.034
Caco-2 permeability	Caco2_Wang	reg	182	–	Spearman 0.587 [0.463, 0.690]	0.686±0.211

**Table 6:** CADT on the DAVIS cold-target split (5168 pairs, 318 actives at $pK_{d}\geq 7$). Load, fraction sent to docking; active recall, strong binders retained; EF, enrichment factor over docking everything.

Stage	Docked (*n*)	Load	Active Recall	Hit Rate	EF
Dock everything (reference)	5168	100%	1.000	0.062	1.00
CADT affinity-confidence gate	710	13.7%	0.610	0.273	4.44
CADT + ADMET (hard filter)	102	2.0%	0.151	0.471	7.65

**Table 7:** Component ablation of the triage cascade at a matched docking budget per split (budgets: random k=763, cold-target k=710, cold-drug k=3641).

Selection Strategy	Random		Cold-Target		Cold-Drug	
	Recall	EF	Recall	EF	Recall	EF
Affinity only (μ)	0.850	5.74	0.610	4.44	0.840	1.22
+uncertainty (μ+σ)	0.848	5.72	0.610	4.44	0.829	1.21
+applicability domain	0.850	5.74	0.610	4.44	0.851	1.24
+soft-ADMET score	0.865	5.84	0.610	4.44	0.811	1.18
Full CADT	0.848	5.72	0.610	4.44	0.654	0.95

**Table 8:** Operating points as the rejection threshold τ is swept.

Split	τ	Load	Confidence Gate		Affinity Only	
			Recall	EF	Recall	EF
Random	5.5	33.3%	0.965	2.89	0.968	2.90
	6.0	14.8%	0.848	5.72	0.850	5.74
	6.5	7.2%	0.625	8.72	0.628	8.77
	7.0	3.3%	0.381	11.70	0.384	11.79
Cold-target	5.5	34.9%	0.881	2.52	0.890	2.55
	6.0	13.7%	0.610	4.44	0.610	4.44
	6.5	5.2%	0.412	7.91	0.415	7.97
	7.0	2.4%	0.233	9.62	0.239	9.88
Cold-drug	5.5	74.1%	0.714	0.96	0.869	1.17
	6.0	68.6%	0.654	0.95	0.840	1.22
	6.5	65.9%	0.620	0.94	0.814	1.24
	7.0	64.8%	0.609	0.94	0.811	1.25

**Table 9:** Spending a fixed docking budget three ways: a hard ADMET filter, affinity alone, or a soft affinity–developability score.

Split	Budget	Hard ADMET		Affinity-Only		Soft Score	
		Recall	EF	Recall	EF	Recall	EF
random	113	0.167	7.62	0.290	13.24	0.279	12.71
cold-target	102	0.151	7.65	0.201	10.20	0.195	9.88
cold-drug	758	0.077	0.54	0.269	1.88	0.260	1.82

**Table 10:** Measured docking cost on six withheld kinases.

Target	PDB	Redock RMSD	All 68	Gated	Saving
BRAF V600E	3OG7	0.57 Å	15.8 h	2.3 h	85.7%
GSK3β	6Y9S	0.78 Å	25.1 h	2.9 h	88.4%
VEGFR2	4ASD	1.00 Å	15.8 h	3.5 h	77.7%
AURKC	6GR8	1.22 Å	14.4 h	1.2 h	91.3%
TAOK3	6BDN	2.50 Å	14.6 h	1.9 h	87.0%
LOK	6EIM	2.71 Å	14.3 h	3.2 h	77.9%
All			99.8 h	15.0 h	85.0%

**Table 11:** DAVIS split sizes (seed 1; other seeds differ only by resampling). Pairs, with the number of unique drugs and targets appearing in each subset.

Protocol (Train/Val/Test)	Pairs	Unique Drugs	Unique Targets
Random	18,041/2577/5154	68/68/68	379/379/379
Cold-target	18,020/2584/5168	68/68/68	265/38/76
Cold-drug	17,813/2653/5306	47/7/14	379/379/379

## Data Availability

Publicly available datasets were analysed in this study; no new experimental data were generated. All datasets were obtained through the Therapeutics Data Commons (TDC) [47], an open repository that redistributes each dataset under its original licence, and can be found at https://tdcommons.ai (accessed on 19 July 2026). The complete source code, the dataset identifiers and fixed random seeds needed to regenerate every split, the model settings, the per-compound prediction files underlying each table and figure, and a pinned software environment (Python 3.12; PyTDC 1.0.0, RDKit 2023.09.6, XGBoost 3.2.0, scikit-learn 1.2.2, PyTorch 2.6, NumPy 1.26), are openly available in Zenodo at https://doi.org/10.5281/zenodo.22076715 (accessed on 25 August 2026).

## Abbreviations

The following abbreviations are used in this manuscript:

ADME	Absorption, distribution, metabolism, and excretion
ADMET	Absorption, distribution, metabolism, excretion, and toxicity
AFT	Accelerated failure time
AUPRC	Area under the precision–recall curve
AUROC	Area under the receiver operating characteristic curve
CADT	Confidence-gated affinity–ADMET docking triage
CI	Concordance index
CNN	Convolutional neural network
DTA	Drug–target affinity
EF	Enrichment factor
GPU	Graphics processing unit
MAE	Mean absolute error
MD	Molecular dynamics
ML	Machine learning
MSE	Mean squared error
RMSE	Root mean squared error
TDC	Therapeutics Data Commons
